# Deep Oxidative Desulfurization of Dibenzothiophene in Simulated Oil and Real Diesel Using Heteropolyanion-Substituted Hydrotalcite-Like Compounds as Catalysts 

**DOI:** 10.3390/molecules181113691

**Published:** 2013-11-05

**Authors:** Fengli Yu, Rui Wang

**Affiliations:** 1 School of Environmental Science and Engineering, Shandong University, Jinan 250100, China; E-Mail: yufengli123@126.com; 2 College of Resource & Environment Engineering, Jilin Institute of Chemical Technology, Jilin 132022, China

**Keywords:** oxidative desulfurization, heteropolyanion, hydrotalcite-like compounds, hydrogen peroxide

## Abstract

Three heteropolyanion substituted hydrotalcite-like compounds (HPA-HTLcs) including Mg_9_Al_3_(OH)_24_[PW_12_O_40_](MgAl-PW_12_), Mg_9_Al_3_(OH)_24_[PMo_12_O_40_] (MgAl-PMo_12_) and Mg_12_Al_4_(OH)_32_[SiW_12_O_40_] (MgAl-SiW_12_), were synthesized, characterized and used as catalysts for the oxidative desulfurization of simulated oil (dibenzothiophene, DBT, in *n*-octane). MgAl-PMo_12_ was identified as an effective catalyst for the oxidative removal of DBT under very mild conditions of atmospheric pressure and 60 °C in a biphasic system using hydrogen peroxide as oxidant and acetonitrile as extractant. The conversion of DBT was nearly 100%. As a result, because of the influence of the electron density and the space steric hindrance, the oxidation reactivity of the different sulfur compounds in simulated oil followed the order DBT > 4,6-dimethyldibenzothiophene (4,6-DMDBT) > benzothiophene (BT) > thiophene (TH). When the reaction is finished, the catalysts can be recovered from the acetonitrile phase by filtration. The recovered MgAl-PMo_12_ retains nearly the same catalytic activity as the fresh material. Moreover, MgAl-PMo_12_ was found to exhibit an ideal catalytic activity in the oxidative desulfurization of real diesel resulting in a total remaining sulfur content of 9.12 ppm(*w*).

## 1. Introduction

The combustion of sulfur contained in fuels produces sulfur dioxide, which is liable to be converted into sulfate aerosols and lead to a series of air pollution events, including acid rain [1]. Human exposure to sulfur dioxide in the ambient air has been related with increased incidence of respiratory system diseases, irritation of the eyes, nose, and throat, and even lung cancer [2,3]. Moreover, the catalysts applied in motor vehicle exhaust gas treatment systems, are always poisoned by SO_2_ and hence the emissions of nitrogen oxides (NO_x_) and total suspended solids (TSP) increase, so it is a major cause of urban environmental problems. Therefore, the specifications for sulfur in diesel have undergone dramatic revisions in many countries. Since 2006, almost all of the petroleum-based diesel fuel available in North America and Europe is an ultra-low sulfur diesel ULSD with substantially lowered sulfur content of 15 ppm. On 1 June 2006, refiners and importers in the United States were required to ensure that at least 80 percent of the volume of the highway diesel fuel they produce or imported was ULSD. In fact, diesel fuel classified as ULSD has been sold on all highways since 1 December 2010. Since 2012, as a further requirement, marine and locomotive diesel fuel must meet the ULSD level [4].

At present, numerous desulfurization methods have been developed. Among them hydrodesulfurization (HDS) has been widely applied to the field of petroleum fuel production around the world for a long time. HDS has shortcomings such as lower desulfurization efficiency, strict reaction conditions and so on, which leads to higher manufacturing costs and higher price of the resulting fuel, therefore, the search for new desulfurization technologies which offer higher efficiency and lower manufacturing costs has become a hot research topic in this field. Oxidative desulfurization (ODS) has the advantages of milder reaction conditions, higher efficiency and easier operation, lower investment, lower operating cost and energy efficient use compared with HDS. ODS methods include two steps: oxidation and extraction/adsorption. Firstly, sulfur compounds are oxidized by an oxidant, next the products are separated from the oil through extraction or adsorption. H_2_O_2_ as well as O_2_, O_3_, K_2_FeO_4_, and nitrogen oxides can be used as oxidants in ODS [5,6,7,8,9,10,11,12,13,14,15,16,17,18,19,20]. Lu *et al*. [19] found that sulfur compounds in fuels can be selectively converted into SO_2_ by mixing the fuel with a small amount of air at around 300 °C and ambient pressure in a continuous-flow reactor packed with catalysts such as Pt/CeO_2_, Cu/CeO_2_, and CuO/ZnO/Al_2_O_3_. Lu *et al*. [20] synthesized a series of CuZnAl oxide-composite catalysts via decomposition of CuZnAl hydrotalcite-like compounds (HTLcs: Cu 37%, Zn 15%, Al 48% mol) at various temperatures and examined their use during the course of aerobic oxidative desulfurization (AODS) of gasoline-range organosulfur compounds in *iso*-octane. They showed that the catalytically relevant properties of HTLcs-derived CuZnAl catalysts and their performance in AODS reactions can be significantly controlled by the HTLcs decomposition temperatures. Song *et al.* [6] found that the oxidation in the presence of molecular oxygen with Fe (III) salts was able to oxidate thiophenic compounds in the fuel. The oxidation reactivity of the sulfur compounds follow the order 2-methylbenzothiophene > 5-methylbenzothiophene > benzothiophene >> dibenzothiophene. Dehkordi *et al.* [7] studied the process of desulfurization of non-hydrotreated kerosene with H_2_O_2_ as oxidant and acetic acid as catalyst and investigated the influences of various operating parameters, including acid to sulfur molar ratio, reaction temperature, and oxidant to sulfur molar ratio on the course of sulfur removal from kerosene. The results showed that the maximum sulfur removal in the oxidative desulfurization system was 83.3% under the optimal experimental conditions. H_2_O_2_ has been used as an ideal and clean oxidant for its less corrosiveness, sole coproduct (H_2_O) and no pollution, which make H_2_O_2_ the best oxidant. In our work, we used H_2_O_2_ as oxidant. Recently, many researchers have applied polyoxometalates as catalyst in ODS [8,9,10,11,12,13,16,17]. Yazu *et al.* [8] studied an oxidative organic liquid-liquid two-phase desulfurization method with phosphotungstic acid as catalyst, H_2_O_2_ as oxidant and acetonitrile as the extraction agent. Phosphotungstic acid exhibited high removal of sulfur compounds from diesel with a sulfur removal rate of 96.36%. Li *et al*. [21] synthesized four surfactant-type polyoxometalate-based ionic liquids (SPILs), which have been investigated for their oxidative desulfurization properties. SPILs were very promising for the desulfurization of DBT in a model oil using H_2_O_2_ as the oxidant. Supported mechanism and kinetics studies on the catalytic activity of SPIL [(n-C_8_H_17_)_3_NCH_3_]_3_{PO_4_[MoO(O_2_)_2_]_4_} revealed that the oxidative desulfurization of organosulfur compounds presented pseudo first-order kinetics. Then, they synthesized a series of POM-based hybrid materials: phosphotungstic acid-supported ceria (HPW-CeO_2_) [22]. The catalyst was very efficient on the removal of DBT using H_2_O_2_ as the oxidant under mild reaction conditions, and could reach a sulfur removal of 99.4%. In our previous research, H_3_PW_12_O_40_, Cs_2*.*5_H_0*.*5_PW_12_O_40_, H_3_PWMo_11_O_40_, H_3_PW_3_Mo_9_O_40_, H_3_PW_6_Mo_6_O_40_ were synthesized and applied to ODS as catalysts [10,11]. H_3_PW_6_Mo_6_O_40_ was applied to the oxidative removal of thiophene, where it showed the more efficiency and easier recovery compared with the five heteropolyacids. H_3_PW_6_Mo_6_O_40_ was dissolved in acetonitrile after the reaction, and then the acetonitrile was separated at 85 °C by distillation. H_3_PW_6_Mo_6_O_40_, sulfone and H_2_O were left in the reactor. In addition, by adding KCl, a precipitate of K_3_PW_6_Mo_6_O_40_ was observed in the reactor. After filtration, the K_3_PW_6_Mo_6_O_40_ was dissolved in deionized water at 70 °C and H_3_PW_6_Mo_6_O_40_ was obtained through ionic exchange with H-type cation exchange resin. However, this process is too complex to be used to industrial applications. We can get good desulfurization efficiency with a single heteropolyacid as catalyst. The heteropolyacid is dissolved in an extractant as a homogeneous catalyst which could not be separated directly after ODS reaction, and hence cannot be used widely in industry. Our works have focused on looking for a kind of catalyst which is not soluble in the oil phase or acetonitrile phase. In our group, a new multi-walled carbon nanotube (MWNT)-supported catalyst (Cs_2.5_H_0.5_PW_12_O_40_/MWNT) has been developed for ODS [12]. Through our experiments [12], we showed that Cs_2.5_H_0.5_PW_12_O_40_/MWNT was an effective catalyst for ultra-low sulfur diesel production by ODS. When the reaction was finished, Cs_2.5_H_0.5_PW_12_O_40_/MWNT can be recovered from the acetonitrile phase by simple filtration.

In this paper, we report on the preparation of some heteropolyanion substituted hydrotalcite-like compounds (HPA-HTLcs), including Mg_9_Al_3_(OH)_24_[PW_12_O_40_](MgAl-PW_12_), Mg_9_Al_3_(OH)_24_ [PMo_12_O_40_](MgAl-PMo_12_) and Mg_12_Al_4_(OH)_32_[SiW_12_O_40_](MgAl-SiW_12_), which were then used as catalysts for the oxidative removal of sulfur compounds from simulated oil and diesel in an oil/acetonitrile biphasic system.

## 2. Results and Discussion

### 2.1. Characterization of the Catalysts

**Figure 1:**
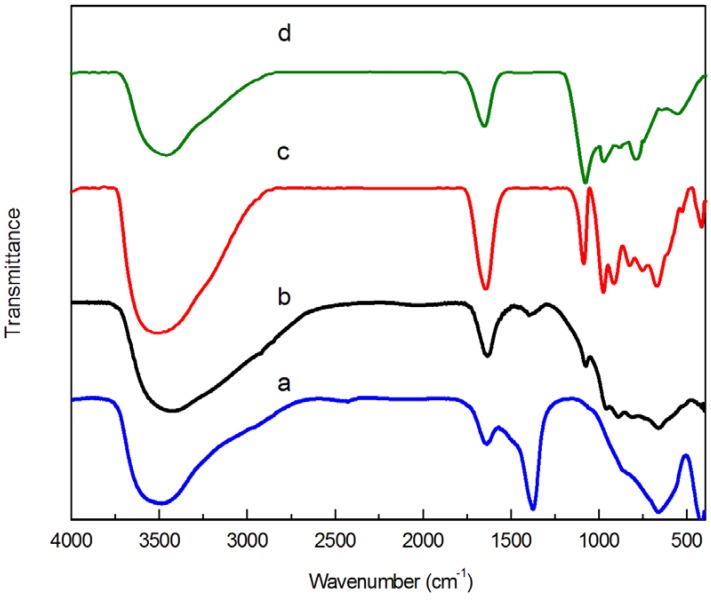
FTIR spectra of samples: (**a**) MgAl-SiW_12_; (**b**) MgAl-PW_12_; (**c**) MgAl-CO_3_; (**d**) MgAl-PMo_12_.

Figure 1 shows the FTIR spectra of Mg_3_Al(OH)_8_CO_3_(MgAl-CO_3_), MgAl-PW_12_, MgAl-SiW_12_ and MgAl-PMo_12_. The band around 1,374 cm^−1^ is considered as the characteristic vibration band which is generated by the antisymmetric stretching vibration of the split peaks of CO_3_^2−^. After the ion exchange reaction, this band of 1,374 cm^−1^ is reduced gradually until it disappears, which showed that the anion in MgAl-CO_3_ has been completely replaced by the heteropolyanion in MgAl-SiW_12_ (a) or MgAl-PMo_12_ (d). There was a MgAl-PW_12_ (b) peak at 1,374 cm^−1^. This means that the heteropolyanion cannot completely replace CO_3_^2−^ in that process. Meanwhile, it shows that four characteristic bands between 1,100 and 700 cm^−1^ were observed in Figure 1 for each catalyst. The key FTIR bands of HPA-HTLcs are the X-O_a_, M=O_d_, M-O_b_-M and M-O_c_-M ones shown in Table 1, respectively. The results indicate that all of the catalysts maintain their Keggin structures [23,24].

**Table 1:** IR spectra band of HPA-HTLcs (cm^−1^).

Sample	X-O_a_	M=O_d_	M-O_b_-M	M-O_c_-M
MgAl-PW_12_	1,073	956	888	808
MgAl-PMo_12_	1,069	959	872	778
MgAl-SiW_12_	1,072	961	902	811

### 2.2. Comparison of the Catalytic Activity of Different HPA-HTLcs

DBT was the hardest to remove sulfur containing compounds in diesel in the primary HDS, so in this paper, DBT was chosen as the typical sulfur component for the study of ODS. DBT was dissolved in *n*-octane to prepare a simulated oil with a sulfur concentration of 500 ppm(*w*). The oxidative desulfurization reaction conditions are as follows: temperature 60 °C, O/S molar ratio 20, the amount of catalyst was 1% (*w*) of *n*-octane. Under the experimental conditions, the DBT is directly converted into the corresponding DBT sulfone [9,10,11,12].

From Table 2, the order of catalytic activity is MgAl-PMo_12_ > MgAl-PW_12_ > MgAl-SiW_12_. This shows that MgAl-PMo_12_ has the best catalytic activity, and the DBT conversion reached almost 100%. Obviously, the catalytic activity of MgAl-PMo_12_ and MgAl-PW_12_ were higher than that of MgAl-SiW_12_. This fact suggested that phosphorus as central atom had higher catalytic activity than silicon. The catalytic activity of MgAl-PMo_12_ was higher than that of MgAl-PW_12_, because of the different activity of the coordinated atoms, meaning that the activity of molybdenum as a coordinated atom is higher than that of tungsten.

**Table 2:** Investigation of desulfurization catalyzed by different catalysts.

Entry	Catalyst	DBT conversion (%)
1	MgAl-PW_12_	80.46
2	MgAl-PMo_12_	100
3	MgAl-SiW_12_	76.76

### 2.3. Oxidative Desulfurization of Simulated Oil with MgAl-PMo as Catalyst

According to the foregoing results, MgAl-PMo_12_ was chosen for further experiments on the effects of several operational factors, including the pre-reaction time of H_2_O_2_ and the catalyst, O/S molar ratio, temperature, and the dosage of catalyst.

#### 2.3.1. Effect of the Hydrogen Peroxide Pre-Reaction Time and the Catalyst

**Figure 2:**
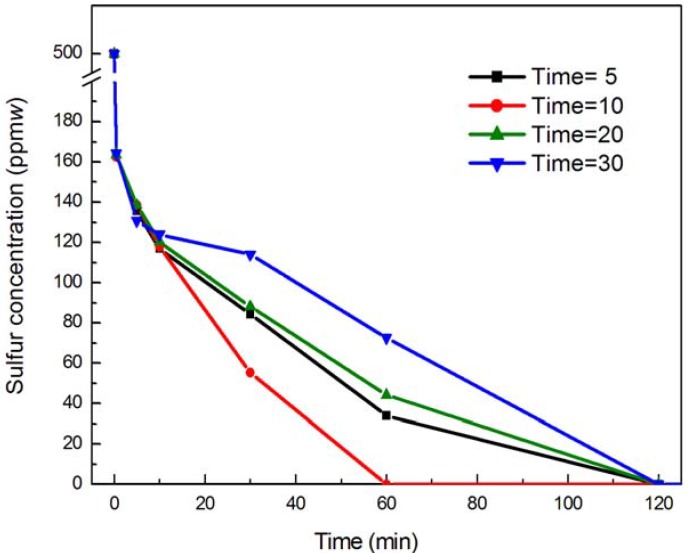
The effect of the pre-reaction time of H_2_O_2_ and MgAl-PMo_12_. Experimental conditions: temperature 60 °C; O/S molar ratio 20; the amount of catalyst used was 1% the mass of *n*-octane; and DBT (S: 500 ppm(*w*) in *n*-octane).

In experiments, at first the MgAl-PMo_12_ catalyst was mixed with 30% aqueous peroxide in a 5 mL test tube. A few minutes later, the mixture was added into the reaction kettle in which the simulated oil and acetonitrile were added. The influence of the pre-reaction time on the DBT conversion was given in Figure 2. With a pre-reaction time from 5 min to 10 min, the conversion of DBT increased. The reaction of H_2_O_2_ and catalyst was not complete because of the short pre-reaction time, which resulted in the low conversion rate in the first stage.

After 60 min, the DBT removal rate was observed to increase from 93.79% to 99.03% as the pre-reaction time was increased from 5 min to 10 min. However, when the pre-reaction time was more than 10 min, the conversion of DBT decreased because of the decomposition of H_2_O_2_. The decomposition of H_2_O_2_ is mainly due to the long pre-reaction time, and the metal ion also has an increased influence on this decomposition. As a whole, the best pre-reaction time can be considered as 10 min.

#### 2.3.2. Effect of Temperature on ODS of Simulated Oil

**Figure 3:**
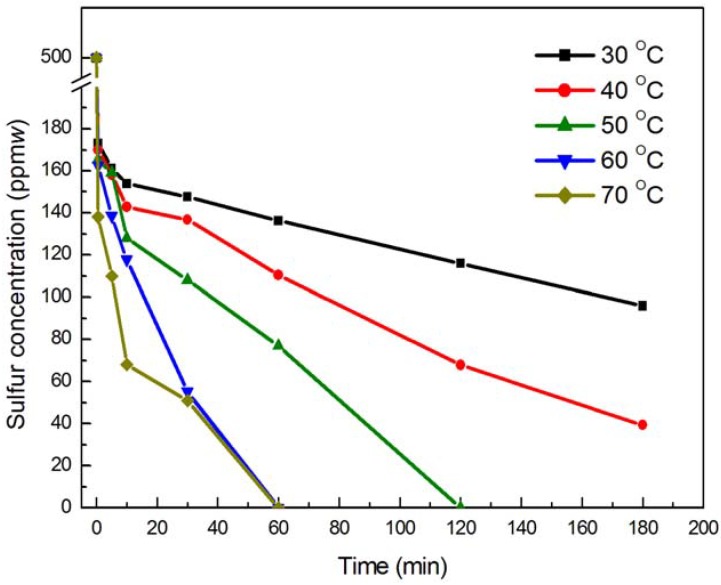
The effect of different temperatures on ODS of simulated oil. Experimental conditions: pre-reaction time 10 min; O/S molar ratio 20; the amount of catalyst used was 1% the mass of *n*-octane; and DBT (S: 500 ppm(*w*) in *n*-octane).

Acetonitrile was used as the extraction solvent in the reaction system. The maximum temperature was 70 °C due to the 81.1 °C boiling point of acetonitrile. The DBT oxidation results with different reaction temperatures under the same conditions are shown in Figure 3.

This data shows that the rate of reaction obviously increases when the temperature is increased. As the temperature increased from 30 °C to 70 °C, the sulfur content of the simulated oil decreased from 147.64 ppm(*w*) to 50.88 ppm(*w*) after 30 min of reaction. At 30 °C and 40 °C, the desulfurization efficiency was 80.82% and 92.13% after 180 min, and the sulfur content in the *n*-octane was 95.89 ppm(*w*) and 39.37 ppm(*w*), respectively. When the temperature went from 50 °C to 70 °C, the desulfurization efficiency was nearly 100% after 120 min of reaction. Consequently, the optimum temperature can be determined as 60 °C.

#### 2.3.3. Effect of O/S Molar Ratio 20 on ODS of Simulated Oil

**Figure 4:**
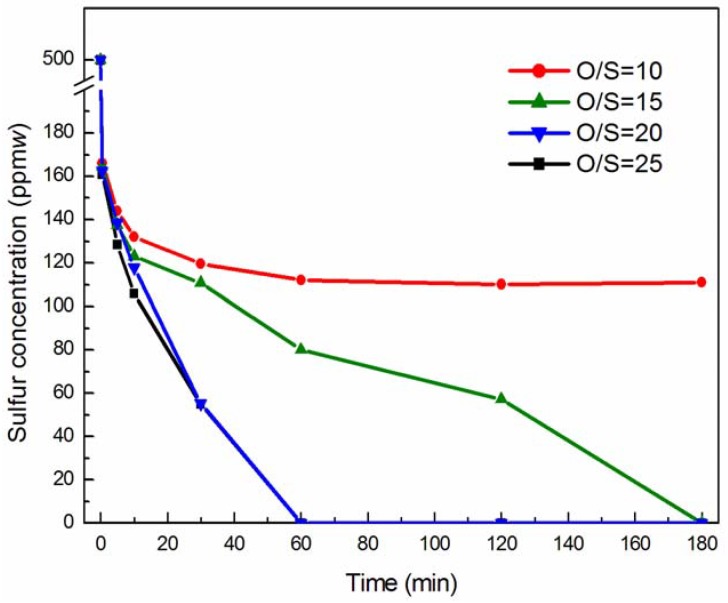
The effect of different O/S molar ratio on ODS of simulated oil. Experimental conditions: pre-reaction time 10 min; temperature 60 °C; the amount of catalyst used was 1% the mass of *n*-octane; and DBT (S: 500 ppm(*w*) in *n*-octane).

The results in Figure 4 showed that the sulfur content conversion was different at different O/S molar ratios. The conversion increased with a high quantity of H_2_O_2_. The DBT conversion increased from 76.07% to 89.01% when the molar ratio of O/S increased from 10 to 25 after reaction for 30 min. When the O/S molar ratio exceeded 10, the DBT conversion was nearly 100% after 180 min. The two DBT conversion rate curves almost coincided with the little change in the sulfur concentration when the molar ratio of O/S was 20 or 25 in Figure 3. This showed that the dosage of H_2_O_2_ was excessive at O/S 25. Considering the cost of H_2_O_2_ in the actual reaction, the molar ratio of O/S 20 was considered proper.

#### 2.3.4. Effect of Different Amounts of Catalyst

**Figure 5:**
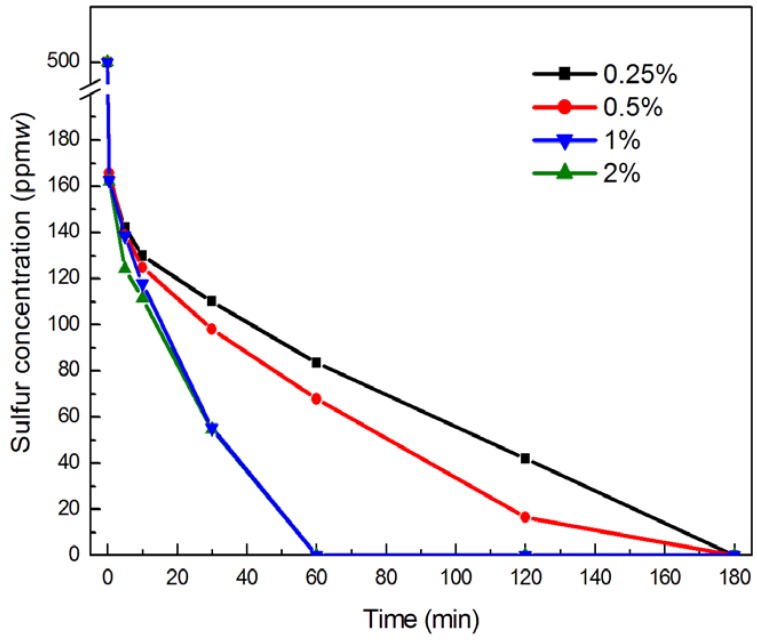
Effect of different amounts of catalyst on ODS of simulated oil. Experimental conditions: temperature 60 °C; O/S molar ratio 20; pre-reaction time 10 min; and DBT (S: 500 ppm(*w*) in *n*-octane).

The influence on the dosage of catalysts on the sulfur removal is given in Figure 5. As the dosage of catalysts increased, the conversion increased. This is due to the increased amount of catalyst in the acetonitrile, which increased the amount of catalytic intermediates produced by the reaction of H_2_O_2_ and catalyst and increased the catalyst activity, which favors the oxidation of DBT. As the dosage of catalyst increased from 0.25% (*w*) to 1% (*w*), the sulfur content conversion increased, as shown in Figure 5. When the dosage of catalyst increased to 2%(*w*) there is no change in the desulfurization efficiency. From the results, the optimum amount of catalyst can thus be determined as 1% (*w*) of *n*-octane.

### 2.4. The Recycle of the MgAl-PMo_12_ Catalyst

After the reaction, the catalyst was precipitated in the reactor, washed with acetonitrile, and filtered. Then, it was dried overnight in an air oven at 80 °C. Under similar conditions, after the 5th recovery of MgAl-PW_12_, the DBT conversion was nearly 99.82% after 120 min, which was quite close to the result obtained with fresh catalyst. The average recovery rate of MgAl-PW_12_ was 89.38%.

### 2.5. Catalyst Effect of Different Sulfur Compounds

**Figure 6:**
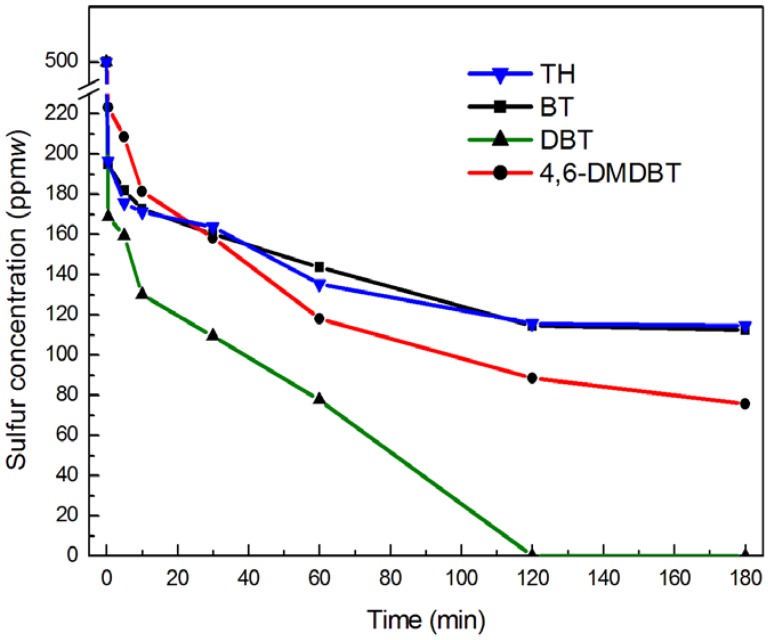
Catalyst effect of different sulfur compounds under the same conditions. Experimental condition: temperature 60 °C; O/S molar ratio 20; pre-reaction time 10 min; the amount of catalyst used was 1% the mass of *n*-octane; and different sulfur compound (S: 500 ppm(*w*)) in *n*-octane.

Under the same conditions, the desulfurization efficiency of different sulfur compounds is shown in Figure 6. It was clear that the conversion rate of DBT in simulated oil could reach nearly 100% after 120 min. Thus, the catalytic activity of the different sulfur compounds was in the order of DBT > 4,6-dimethyldibenzothiophene (4,6-DMDBT) > benzothiophene (BT) > thiophene (TH). The electron densities on the sulfur atoms of 4,6-DMDBT, DBT, BT and TH were 5.760, 5.758, 5.739 and 5.696, respectively [25]. When the electron density on a sulfur atom was higher, the catalytic activity was higher [22]. The electron density of the sulfur atom in TH is the lowest, which led to the lowest catalytic activity towards TH, but it was different in the order of DBT and 4,6-DMDBT. In fact, the spatial steric hindrance is another important factor in oxidative desulfurization [22]. The new bonds between oxygen and sulfur atoms would be hindered by the alkyl groups of the 4 and 6 position in 4,6-DMDBT during oxidative desulfurization, therefore, the activity of DBT was higher than that of 4,6-DMDBT in ODS.

### 2.6. The Effect of Sulfur Removal in Diesel Oil

The optimum experiment conditions were 60 °C, O/S mole ratio 20, catalyst 1% (*w*), pre-reaction time 10 min, and a 1:1 volume ratio of acetonitrile and diesel. After reaction for 180 min, the sulfur compounds in diesel decreased from 493 ppm(*w*) to 107 ppm(*w*), and the sulfur removal rate was 78.30%. When double the amount ao fresh acetonitrile to diesel was used in the ODS, the sulfur concentration further decreased to 34.8 ppm(*w*). After oxidative desulfurization, diesel was extracted with another amount of fresh solvent, and then washed by distilled water, after which the sulfur concentration in diesel was 9.12 ppm(*w*).

### 2.7. The Mechanism of the ODS Process

**Figure 7:**
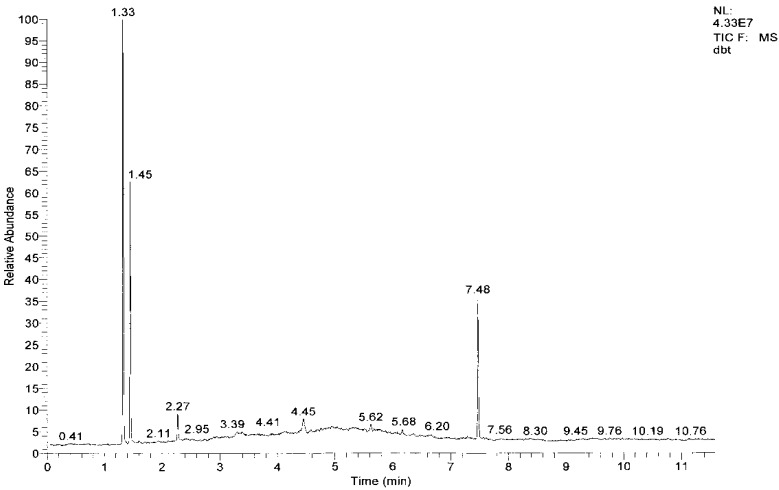
GC-MS analysis for sulfur compounds of acetonitrile after ODS. Retention times: acetonitrile, 1.33 min; *n*-octane, 1.45 min; DBT, 5.62 min; DBT sulfone, 7.48 min.

**Scheme 1:**
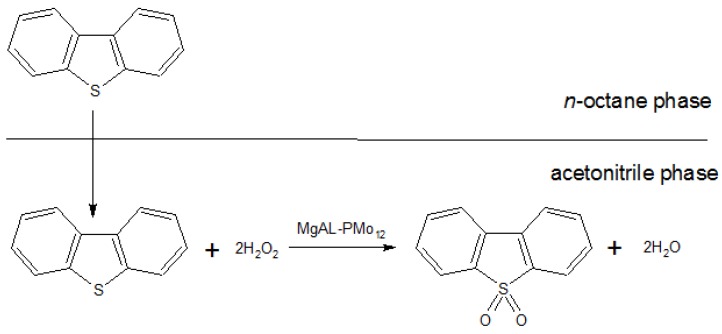
The DBT desulfurization process.

Because hydrogen peroxide can be dissolved in acetonitrile, additional hydrogen peroxide as an oxidant was put into acetonitrile, and DBT transferred from *n*-octane to acetonitrile. DBT was oxidized to DBT sulfone. The polarity of the DBT sulfone is higher than that of DBT, and hence the former remained in the acetonitrile phase. With the aid of GC-MS, the product of DBT oxidation by H_2_O_2_ in the acetonitrile phase after 120 min was confirmed to consist only of the corresponding sulfone species (Figure 7). Hence, the overall reaction equation can be expressed as shown in Scheme 1.

## 3. Experimental

### 3.1. Catalyst Preparation

The HPA-HTLcs were synthesized using ion-exchange to intercalate heteropolyanions into the layers of hydrotalcite-like compounds (HTLcs) as described in the literature [16]. MgAl-CO_3_ was prepared by a sequential precipitation technique—the precipitants (2.0 M NaOH, 1.0 M Na_2_CO_3_) and metal nitrate solutions (6.0 M Mg(NO_3_)_2_, 3.0 M Al(NO_3_)_2_) were added to distilled water drop by drop under vigorous stirring, then heated to 40 °C, and then titrated with pH 10 of the solution at a rate of one drop per second. Normally, this took nearly one and a half hours. The slurry was aged at 65 °C for 18 h, pump filtered, and washed with distilled water until the filtered liquid was neutral. Next, the pressed cake was dried overnight in an air oven at 80 °C.

MgAl-CO_3_ prepared by the above-mentioned method was dispersed in distilled water, until fully swollen. An excess amount of heteropolyanions was slowly added to the solution, while dilute nitric acid was used to maintain 2 < pH < 4. Ion exchange occurred in the reaction mixture that was maintained at 65 °C for about 10 h, pump filtered, and washed with distilled water until the filtered liquid was neutral. Finally, the sample was dried overnight in an air oven at 80 °C.

### 3.2. Catalyst Characterization

The infrared spectra (IR) of all the catalysts were recorded on an Avatar 370 spectrometer (Thermo Electron Corporation, Madison, WI, USA).

### 3.3. Oxidative Desulfurization of Simulated Fuel Oil

Simulated oil was prepared by dissolving DBT, TH, BT and 4,6-DMDBT in *n*-octane to a sulfur concentration of 500 ppm(*w*). At first, the catalyst was dissolved in 30% aqueous peroxide for 10 min (the H_2_O_2_ and catalyst pre-reaction time). In a typical experimental setup, simulated oil (60 mL), acetonitrile (60 mL), an amount of H_2_O_2_ based on the desired O/S molar ratio and an amount of catalyst as a percent of the mass of *n*-octane, were placed in a reaction kettle and stirred at 60 °C under the atmospheric pressure. Lastly, the catalyst and 30% aqueous peroxide were added to the reaction kettle. DBT content in *n*-octane phase before and after reaction were analysed by GC-FID (Shandong Lunan Ruihong Chemical Engineering Instruments Co., Ltd, Tengzhou, China) using a capillary column (DB-1, 30 m × 0.32 nm, 1.0 μm film thickness). The temperatures for the GC-FID tests were set as 334 °C for both injector and the detector, and 280 °C for the oven. The concentration of DBT was quantified by the external standard method. The content of other sulfur compounds in the *n*-octane phase was determined by a WK-2E micro-Coulomb instrument (Jiangsu Jiangfen Instruments Co., Ltd, Jiangyan, China). The temperatures for the total sulfur tests were set at 700 °C for the stable chamber, 850 °C for combustor, and 650 °C for injector. The sulfur concentration of sample was determined by the sulfur standard sample. The product derived from DBT oxidation by H_2_O_2_ was analysed using an Agilent HP-6890N/MS-5793 GC-/MS analyser (Agilent Technologies, Palo Alto, CA, USA). The GC-MS conditions were as follows: injector 290 °C; split ratio 50; column DB-1 MS; oven temperature program, 150 °C for 1 min, 20 °C/min to 280 °C, and hold for 30 min.

### 3.4. Oxidative Desulfurization of Diesel

Oxidative desulfurization of diesel (sulfur 493 ppm(*w*)) was conducted under the same typical experimental conditions as the oxidation of the simulated fuel oil using MgAl-PMo_12_ as catalyst. The catalyst was 1% (*w*) of the diesel oil, the amount of H_2_O_2_ corresponded to an O/S mole ratio of 20, and a constant temperature of 60 °C was used. After the reaction, the kettle was cooled down to room temperature. The oil phase was separated and washed with deionized water. The total amount of sulfur concentration in the diesel was measured using a WK-2E micro-Coulomb instrument. The temperatures for the total sulfur tests were set at 700 °C for the stable chamber, 850 °C for combustor, and 650 °C for injector. The sulfur concentration of the sample was determined using a sulfur standard sample.

## 4. Conclusions

In summary, heteropolyanion substituted hydrotalcite-like compounds were synthesized by an ion-exchange method. It was shown that the catalytic activity of MgAl-PMo_12_ and MgAl-PW_12_ was better than that of MgAl-SiW_12_, due to the central atom of phosphorus in HPA-HTLcs being better than silicon. Then, the coordinated molybdenum atoms were another important factor. MgAl-PMo_12_ showed the best catalytic activity, and the sulfur removal from the simulated oil can reach 100% in 120 min. The most optimum experimental condition was suggested to be temperature 60 °C, O/S mole ratio 20, amount of catalyst used 1% of the mass of *n*-octane, and pre-reaction time 10 min. Moreover, the catalyst can be reclaimed by filtration and reused like fresh one. Under the above conditions, the oxidative reactivity of different sulfur compounds decreased according to the order DBT > 4,6-DMDBT > BT > TH. Under the optimum experimental conditions, the sulfur content in diesel decreased from 493 ppm(*w*) to 9.12 ppm(*w*). In a word, this study clearly shows that HPA-HTLcs are an effective catalyst for ODS and they seem to be a promising alternative for sulfur removal in the petroleum industry.
